# Treatment outcomes of single-visit versus multiple-visit non-surgical endodontic therapy: a randomised clinical trial

**DOI:** 10.1186/s12903-015-0148-x

**Published:** 2015-12-19

**Authors:** Amy Wai-Yee Wong, Cissy Sung-Chi Tsang, Shinan Zhang, Kar-Yan Li, Chengfei Zhang, Chun-Hung Chu

**Affiliations:** 1 Faculty of Dentistry, The University of Hong Kong, Hong Kong, China; 2 University Health Service, The University of Hong Kong, Hong Kong, China

**Keywords:** Deferred endodontic treatment, Single visit, Clinical trial, Root canal therapy

## Abstract

**Background:**

Clincians have been providing single-visit and multiple-visit endodontic treatments for their patients. This study aims to compare the success rate, prevalence of postoperative pain and chairside time of single-visit and multiple-visit endodontic treatments.

**Method:**

Patients who required primary endodontic treatment in a university dental clinic were randomly allocated to two general dentists for single-visit or multiple-visit treatments using the same materials and procedures. Ni-Ti rotary files were used to prepare the root canals, which were subsequently obturated with a core-carrier technique. The chairside time was recorded. The treated teeth were followed up every 6 months on clinically signs and symptoms including pain, tenderness to percussion, sinus tract, mobility and abscess. Periapical radiographs were taken to assess periapical pathology. Successful treatments were neither clinical signs/symptoms noted nor radiographic periapical pathology found postoperatively.

**Results:**

A total of 220 teeth from patients aged 46.4 ± 14.1 were followed up for at least 18 months. The mean (±SD) follow-up period was 29.4 ± 9.3 months. The success rates of single-visit and multiple-visit treatments were 88.9 and 87.4 %, respectively (*p* = 0.729, effect size odds ratio = 1.156). Maxillary teeth had odds ratios of 3.16 (95 % CI: 1.33 to 7.46; *p* = 0.009) and absence of preoperative apical periodontitis had odds ratios of 4.35 (95 % CI: 1.43 to 13.24; *p* = 0.010) were identified from logistic regression as having a higher success rate. The average chairside times of single-visit and multiple-visit treatments were 62.0 and 92.9 min, respectively (mean difference = −30.9, 95 % CI: −39.4 to −22.4, *p* < 0.001, effect size odds ratio = −0.996). Single-visit and multiple-visit treatment had no significant difference in the prevalence of postoperative pain within 7 days (21 and 12 %, *p* = 0.055, effect size odds ratio = 2.061) and after at least 18 months (0.9 and 1.0 %, *p* > 0.999, effect size odds ratio = 0.879).

**Conclusions:**

The success rate and prevalence of postoperative pain of single-visit or multiple-visit treatment had no significant difference. The chairside time for single-visit treatment was shorter than multiple-visit treatment.

**Trial registration:**

Clinical Trials (WHO) ChiCTR-IOR-15006117 registered on 20 March 2015.

## Background

Endodontic treatment used to take multiple visits to complete, with one of the main reasons for this being that it requires a considerable amount of time to complete the treatment [1]. Multiple-visit root canal treatment is well accepted as a safe and common therapy. However, the rationales for multiple-visit endodontic treatment are being questioned. A systematic review [1] found no significant differences in antimicrobial efficacies have been reported between single-visit and multiple-visit treatments. In addition, the use of contemporary endodontics techniques and equipment such as magnifying devices, electronic apex locators, engine-driven rotary nickel titanium files and so forth not only increases the success rate of endodontic treatment but also shortens the time needed for the treatment [2]. Endodontic treatment may therefore be completed in a single visit.

Surveys found many general dentists and endodontists preferred to perform root canal treatment in a conventional way, i.e., multiple visits [3–7]. A review found patients undergoing a single visit experienced a higher frequency of swelling and were more likely to take painkillers [8]. However, a meta-analysis found no significant difference in postoperative complications between single-visit and multiple-visit endodontic treatment [1]. There are numerous advantages to completing root canal therapy in a single appointment, such as there is no risk of flare-up induced by leakage of the temporary seal between appointments and materials needed for separate visits are saved [9]. A successful clinical outcome is commonly regarded as absence of signs and symptoms and no radiological evidence of periapical pathology [10–13].

The on-campus University Health Service dental clinic was established to provide dental services to full-time and part-time students, staff and their dependents at the University of Hong Kong [14]. The dental clinic provides comprehensive general dental care, including primary non-surgical endodontic treatment for eligible patients. The quality of dental services is regularly monitored by an annual patient satisfaction survey for continual improvement [15]. The aim of this study was to evaluate the treatment outcomes of non-surgical primary endodontic treatment root canal therapy using either single-visit or multiple-visit endodontic treatment performed by general dentists in the on-campus dental clinic.

## Method

### Hypotheses tested and outcomes measured

Three null hypotheses were tested in this study. First, there would be no difference between the success rate of single-visit and multiple-visit non-surgical endodontic treatment. Second, there would be no difference between the prevalence of postoperative pain for single-visit and multiple-visit non-surgical endodontic treatment. Third, there would be no difference between the chairside time used for single-visit and multiple-visit non-surgical endodontic treatment. The primary outcome measured was the success of endodontic treatment which is no clinical sign and symptom and no radiographic radiolucency in the follow-up examination. Another outcome measured was the total chairside time spent on completion of endodontic treatment by a single visit and multiple visits. The secondary outcome measured was the prevalence of postoperative pain after 7 days and at the final evaluation (18 to 45 months after treatment) for the single-visit and multiple-visit treatment.

### Patient recruitment

**Fig. 1:**
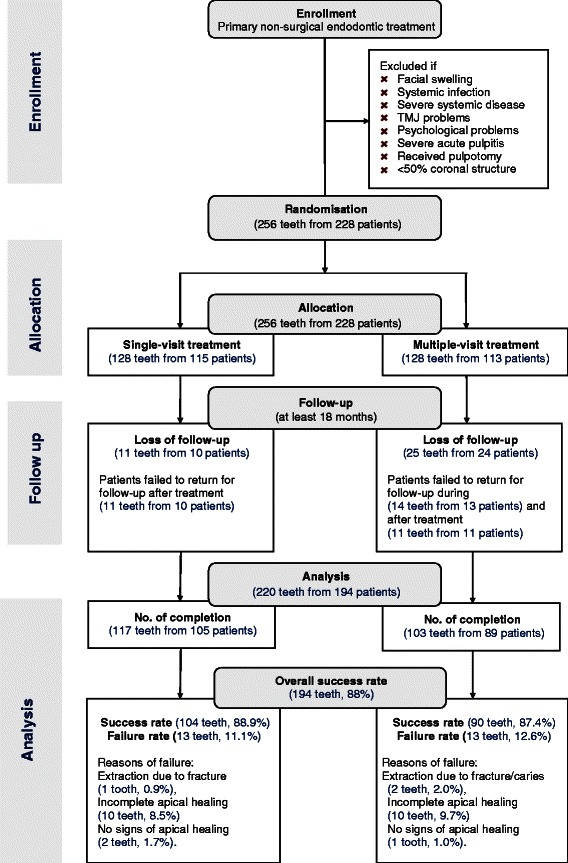
Flow chart of the clinical trial

The study was approved by the Institutional Review Board of the University of Hong Kong/Hospital Authority Hong Kong West Cluster (HKU UW 09–303). The clinical trial was registered in the Chinese Clinical Trial Registry of the World Health Organization (ChiCTR-IOR-15006117). The clinical trial was 4 years. Patient recruitment was implemented for the first 30 months so that the participants would be followed up for at least 18 months. Patients after who were generally healthy, required primary non-surgical endodontic treatment and agreed to return for follow-up via the Health Service Dental Clinic of the University of Hong Kong were invited to participate in the study. Furthermore, the participating patients had no history of periodontitis, and the tooth that required primary endodontic treatment was periodontally healthy. Teeth with pulpotomy were not accepted, and at least half of the coronal structure had to be remaining. The protocol of the study was explained to participants and consent was obtained. Patients who had severe acute pulpitis with facial swelling or systemic infection, severe systemic disease, increased stress on the temporomandibular joint musculature or increased psychological stress were excluded from this study (Fig. 1).

### Group assignment

The participating patients were randomly assigned by the receptionist for endodontic treatment. The treated teeth were randomly assigned to either single-visit or multiple-visit treatments using the random-number generating function of a calculator. If the patient needed more than one endodontic treatment, the randomised allocation was performed on every tooth required treatment. A number unknown to the operators and the independent assessor was given to each treated tooth for clinical and radiographic assessment, data entry and analysis.

### Sample size calculation

For sample size calculation, it has been estimated the success rate of primary non-surgical endodontic treatment was 88 % [12]. A difference in the success rate by at least 10 % between single-visit and multiple-visit endodontic treatments was considered clinically significant and statistically achievable. The estimated sample size was 102 for each treatment group based on the power of the study set at 80 % (β = 0.20) and with α = 0.05 as the significance level. We estimated the dropout rate would be 20 %, and thus, at least 256 teeth with at least 128 teeth per group were required at the baseline for analysis.

### Clinical procedure

The two general dentists (A and B) carried out the endodontic treatments. One of them (A) was trained to use a magnifying loupe (2.5x). The two dentists received a calibration workshop prior to this clinical trial to standardise the instrumentation and obturation technique described below. Preoperative periapcal radiographs using a parallel technique were taken. Local anaesthetic was given and rubber dam was used for isolation. The root canals were cleaned and shaped using Ni-Ti rotary files (ProTaper NiTi, Dentsply Maillefer, Ballaigues, Switzerland). A 5.25 % sodium hypochlorite was used for irrigation. The prepared tooth was obturated after shaping and cleaning of the canals if it was in the single-visit group. For those teeth assigned to multiple-visit group, non-setting 5 % calcium hydroxide paste (UltraCal XS, Ultradent, South Jordan, UT, USA) was used as inter-appointment medication. The tooth was temporarily restored with resin-modified zinc oxide and eugenol cement (IRM, LD Caulk Dentsply, Milford, CT, USA) until obturation. The next appointment was scheduled in following week. It could be two to three visits depending on the complexity of the treatment. All teeth were obturated using a core-carrier technique (Thermafil, Dentsply Maillefer, Ballaigues, Switzerland). The total chairside time was recorded by the dental assistant. The treated teeth were restored with silver amalgam or composite resin. Patients were recommended to take a dose of paracetamol 500 to 1000 mg every 4 to 6 h if needed. All patients were reviewed 1 week after obturation, and were advised to have indirect extra-coronal restoration (partial or full veneer) to avoid failure due to extra-coronal leakage or tooth fracture.

### Evaluation

**Fig. 2:**
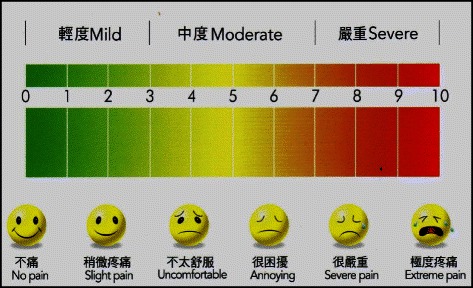
Pain scale score table

The patients were reviewed 1 week after obturation. The treated teeth were clinically examined and reason for clinical failure, if any, was recorded. Clinical signs and symptoms including pain, tenderness on percussion, caries (primary or secondary), defective margin of restoration, mobility, periodontal pocket and soft tissue pathology such as abscess or sinus tract were recorded. If the patient experienced pain or discomfort of treated tooth after obturation, they were asked to rate their pain or discomfort using a pain scale score table (Fig. 2). The pain assessment was adopted from our previous study which measured pain on a 10-point Likert scale, ranging from no pain (score 0) to extreme pain (score 10) [16]. The patients were asked to attend regular follow-ups every 6 months after the endodontic treatment. Periapical radiographs were taken using a parallel technique. The method of radiographic assessment was adopted from Chu and his co-workers (2005) [12]. Signs of any internal or external root resorption were recorded and the periapical conditions were classified as 1) normal—normal appearance of the surrounding osseous structure or 2) apical periodontitis—apical radiolucency observed. Multiple-rooted teeth with different periapical statuses at different roots were classified according to the most severe periapical condition. When doubt existed as to whether pathological periapical conditions were present or not, the case was classified as normal. The method of radiographic assessment for the length and density of the root canal filling were recorded for analysis [16]. The length of the root canal filling were recorded as 1) adequate – filling within 2 mm from radiographic apex, 2) overfilling – filling over radiographic apex or 3) underfilling – filling at least 2 mm short from apex. The density of root canal filling were recorded as 1) adequate – filling uniformly packed without visible voids and canal spaces or 2) inadequate – filling with visible voids or canal spaces. The outcome of the endodontic treatment was classified as a success or a failure. Success was graded when there were no clinical signs/symptoms and no radiographic radiolucency found in the periapical radiograph. The reason for the extraction, in particular for those reasons related to endodontic failure, was recorded. To estimate the reliability of the radiographic assessment, duplicated assessment were performed on around 15 % the patients. The intra-observer agreement and inter-observer agreement for radiographic assessments (complete healing or failure) were then calculated by Kappa statistics.

### Data analysis

The collected data was analysed with the IBM® SPSS® Statistics 21.0 program (IBM SPSS Statistics for Windows, Version 21.0. Armonk, NY: IBM Corp.) by a statistician (SKYL). The intra-observer agreement and inter-observer agreement for radiographic assessments (complete healing or failure) were calculated by Kappa statistics. For the primary treatment outcome (success or failure), multiple logistic regressions were used to assess the relationship between the primary treatment outcome (success or failure) and the treatment (single-visit or multiple-visit) groups, adjusting for other independent variables. The independent variables included: patients’ gender and age, operators (A or B), use of magnifying loupe, arch (maxillary or mandibular), tooth location (anterior or posterior), number of canals of the endodontically treated tooth (single or multiple), presence of preoperative apical radiolucency, presence of C-shaped canal before treatment, presence of periodontal pocket before treatment (≥4 mm pocket), vitality before treatment, mobility before treatment, tooth status of the main opposing tooth, tenderness on percussion before treatment, presence of sinus tract before treatment, presence of pain and pain intensity (0 to 10) before and after treatment, length of root canal filling (adequate, overfilling or underfilling), density of root canal filling (adequate or inadequate) and type of postoperative restoration. All of the independent variables were entered into the model. Backward stepwise procedures were then performed until only variables demonstrating a statistically significant association remained in the final model.

The prevalence of postoperative pain after 1 week and at least 18 months was the secondary outcome evaluated in this study. Chi-square test or Fisher’s exact test was used to compare the proportions between single-visit and multiple-visit groups. The level of statistical significance of all tests was set at 5 %.

The chairside time required for endodontic treatment was the secondary outcome evaluated in this study. Multi-way analysis of covariance (ANCOVA) was used to study the relationship between chairside time and treatment visit group, with the independent variables factored in. The independent variables included: patients’ gender and age, use of magnifying loupe, arch (maxillary or mandibular), tooth location (anterior or posterior), number of canals of the endodontically treated tooth (single or multiple), presence of preoperative apical radiolucency, presence of C-shaped canal before treatment, presence of periodontal pocket before treatment (≥4 mm pocket), vitality before treatment, mobility before treatment, tooth status of the main opposing tooth, tenderness on percussion before treatment, presence of sinus tract before treatment, presence of pain and pain intensity (0 to 10) before treatment. Backward stepwise procedures were used.

## Results

A total of 228 patients with 256 teeth were recruited, and 34 patients with 36 teeth were lost in the follow-up (Fig. 1). The dropout rate was 14.1 %. A total of 194 patients with 220 teeth from aged 46.38 ± 14.06 were followed up for at least 18 months, and the mean (±SD) review period was 29.4 ± 9.3 months. The reason for dropout was patients failed to return for follow up during the treatment or after completion of treatment. Among them, there were 85 male (38.6 %) patients; 117 teeth (53.2 %) were treated with single-visit endodontic treatment; 49 teeth (22.3 %) were anterior teeth; and 89 teeth (40.5 %) had a single-root canal (Table 1). To estimate the reliability, radiographic assessment in around 16 % of the sample (*n* = 36) was duplicated. Kappa values of the intra-observer agreement for the two observers were both 1.000. Kappa value of the inter-observer agreement was 0.911 (standard error = 0.039).

**Table 1:** Independent variables according to treatment group (*n* = 220)

Variable	Category	All cases (*n* = 220)	Single visit (*n* = 117)	Multiple visits (*n* = 103)	*p* value
		No (Col %)	No (Col %)	No (Col %)	
Gender	Male	85 (39 %)	60 (51 %)	25 (24 %)	<0.001*
	Female	135 (61 %)	57 (49 %)	78 (76 %)	
Operator	A	112 (51 %)	56 (48 %)	56 (54 %)	0.335
	B	108 (49 %)	61 (52 %)	47 (46 %)	
Use of loupe	Yes	112 (51 %)	56 (48 %)	56 (54 %)	0.335
	No	108 (49 %)	61 (52 %)	47 (46 %)	
Arch	Maxillary	140 (64 %)	82 (70 %)	58 (56 %)	0.034*
	Mandibular	80 (36 %)	35 (30 %)	45 (44 %)	
Tooth position	Anterior	49 (22 %)	31 (26 %)	18 (17 %)	0.109
	Posterior	171 (78 %)	86 (74 %)	85 (83 %)	
Number of canal	Single	89 (40 %)	55 (47 %)	34 (33 %)	0.035*
	Multiple	131 (60 %)	62 (53 %)	69 (67 %)	
Apical periodontitis	Yes	129 (59 %)	66 (56 %)	63 (61 %)	0.475
	No	91 (41 %)	51 (44 %)	40 (39 %)	
C-shaped canal	Yes	4 (2 %)	2 (2 %)	2 (2 %)	>0.999^a^
	No	216 (98 %)	115 (98 %)	101 (98 %)	
Periodontal pocket	Yes	32 (15 %)	17 (15 %)	15 (15 %)	0.994
	No	188 (85 %)	100 (85 %)	88 (85 %)	
Tooth vitality	Vital	37 (17 %)	20 (17 %)	17 (17 %)	0.907
	Non-vital	183 (83 %)	97 (83 %)	86 (83 %)	
Tooth mobility	Yes	8 (4 %)	2 (2 %)	6 (6 %)	0.151^a^
	No	212 (96 %)	115 (98 %)	97 (94 %)	
Opposing teeth	Missing	6 (3 %)	3 (3 %)	3 (3 %)	0.367^a^
	Sound	153 (70 %)	84 (72 %)	69 (67 %)	
	Filled	43 (19 %)	24 (20 %)	19 (18 %)	
	Crown	18 (8 %)	6 (5 %)	12 (12 %)	
Tender to percussion	Yes	109 (50 %)	49 (42 %)	60 (58 %)	0.015*
	No	111 (50 %)	68 (58 %)	43 (42 %)	
Abscess or sinus tract	Yes	42 (19 %)	20 (17 %)	22 (21 %)	0.422
	No	178 (81 %)	97 (83 %)	81 (79 %)	
Preoperative pain	Yes	80 (36 %)	33 (28 %)	47 (46 %)	0.007*
	No	140 (64 %)	84 (72 %)	56 (54 %)	
Length of root canal filling	Adequate	181 (82 %)	101 (86 %)	80 (78 %)	0.115^a^
	Overfilling	33 (15 %)	15 (13 %)	18 (17 %)	
	Underfilling	6 (3 %)	1 (1 %)	5 (5 %)	
Density of root canal filling	Adequate	203 (92 %)	109 (93 %)	94 (91 %)	0.598
	Inadequate	17 (8 %)	8 (7 %)	9 (9 %)	
Postoperative restoration	Intra-coronal	70 (32 %)	33 (28 %)	37 (36 %)	0.220
	Extra-coronal	150 (68 %)	84 (72 %)	66 (64 %)	
Treatment outcome	Success	194 (88 %)	104 (89 %)	90 (87 %)	0.729
	Failure	26 (12 %)	13 (11 %)	13 (13 %)	
Postoperative pain within 1 week	Yes	37 (17 %)	25 (21 %)	12 (12 %)	0.055
	No	183 (83 %)	92 (79 %)	91 (88 %)	
Postoperative pain at final evaluation (≥18 months)	Yes	2 (1 %)	1 (1 %)	1 (1 %)	>0.999^a^
	No	218 (99 %)	116 (99 %)	102 (99 %)	
Age [Mean (SD)]		46.38 (14.06)	46.80 (15.24)	45.89 (12.65)	0.629
Preoperative pain intensity [Mean (SD)]		1.58 (2.62)	1.01 (2.12)	2.23 (2.97)	0.001*
Chairside Time [Mean (SD)]		76.48 (34.57)	62.03 (23.47)	92.90 (37.77)	<0.001*
Postoperative pain intensity [Mean (SD)]		0.05 (0.48)	0.04 (0.46)	0.05 (0.49)	0.928

*p < 0.05

^a^Fisher’s exact test

The success rate of endodontic treatment was 88.2 % (*n* = 194) in this study. The success rates for the single-visit treatment (*n* = 104) and multiple-visit treatment (*n* = 90) were 88.9 and 87.4 %, respectively (Chi-square test: *p* = 0.729, effect size odds ratio = 1.156). The relationship between the primary treatment outcome (success or failure) was not significantly related to the treatment visit, i.e., single visit or multiple visits (*p* = 0.764) in the full model of the multiple logistic regression adjusted for the aforementioned independent variables (Table 2). Results of the final logistic regression after backward elimination showed that maxillary teeth and absence of preoperative apical radiolucency had higher odds ratios of success (Nagelkerke R^2^ = 0.138). The maxillary teeth had odds ratios of 3.16 (95 % CI: 1.33 to 7.46; *p* = 0.009) with reference to the mandibular teeth. The teeth without apical radiolucency had odds ratios of 4.35 (95 % CI: 1.43 to 13.24; *p* = 0.010) with reference to the teeth with apical radiolucency.

**Table 2:** The effects of treatment visit and other variables on treatment success

Variable	Category	Treatment Outcome		Full model *p*-value	Final model *p*-value
		Success No (Row %)	Failure No (Row %)		
Treatment visit	Single	104 (89 %)	13 (11 %)	0.764	
	Multiple	90 (87 %)	13 (13 %)		
Gender	Male	74 (87 %)	11 (13 %)	0.980	
	Female	120 (89 %)	15 (11 %)		
Use of loupe	Yes	99 (88 %)	13 (12 %)	0.470	
	No	95 (88 %)	13 (12 %)		
Arch	Maxillary	130 (93 %)	10 (7 %)	0.015*	0.009*
	Mandibular	64 (80 %)	16 (20 %)		
Tooth position	Anterior	46 (94 %)	3 (6 %)	0.855	
	Posterior	148 (87 %)	23 (13 %)		
Number of canal	Single	82 (92 %)	7 (8 %)	0.159	
	Multiple	112 (85 %)	19 (15 %)		
Apical periodontitis	Yes	107 (83 %)	22 (17 %)	0.021*	0.010*
	No	87 (96 %)	4 (4 %)		
C-shaped canal	Yes	4 (100 %)	0 (0 %)	0.999	
	No	190 (88 %)	26 (12 %)		
Periodontal pocket	Yes	26 (81 %)	6 (19 %)	0.247	
	No	168 (89 %)	20 (11 %)		
Tooth vitality	Vital	35 (95 %)	2 (5 %)	0.862	
	Non-vital	159 (87 %)	24 (13 %)		
Tooth mobility	Yes	7 (88 %)	1 (13 %)	0.365	
	No	187 (88 %)	25 (12 %)		
Opposing teeth	Missing	6 (100 %)	0 (0 %)	0.804	
	Sound	136 (89 %)	17 (11 %)		
	Filled	36 (84 %)	7 (16 %)		
	Crown	16 (89 %)	2 (11 %)		
Tender to percussion	Yes	95 (87 %)	14 (13 %)	0.128	
	No	99 (89 %)	12 (11 %)		
Abscess or sinus tract	Yes	35 (83 %)	7 (17 %)	0.655	
	No	159 (89 %)	19 (11 %)		
Preoperative pain	Yes	74 (93 %)	6 (8 %)	0.086	
	No	120 (86 %)	20 (14 %)		
Length of root canal filling	Adequate	162 (90 %)	19 (10 %)	0.242	
	Overfilling	28 (85 %)	5 (15 %)		
	Underfilling	4 (67 %)	2 (33 %)		
Density of root canal filling	Adequate	182 (90 %)	21 (10 %)	0.039*	
	Inadequate	12 (71 %)	5 (29 %)		
Postoperative restoration	Intra-coronal	62 (89 %)	8 (11 %)	0.927	
	Extra-coronal	132 (88 %)	18 (12 %)		
Postoperative pain within 1 week	Yes	34 (92 %)	3 (8 %)	0.943	
	No	160 (87 %)	23 (13 %)		
Age [Mean (SD)]		46.07 (14.23)	48.65 (12.75)	0.535	
Preoperative pain intensity [Mean (SD)]		1.64 (2.64)	1.15 (2.46)	0.653	

(Full and final multiple logistic regression) (*n* = 220)

*p < 0.05

The prevalence of postoperative pain after 1 week of the single-visit and multiple-visit were 21 and 12 % (Chi-square test: *p* = 0.055, effect size odds ratio = 2.061), whereas the prevalence of postoperative pain after at least 18 months were 0.9 and 1.0 %, respectively (Fisher’s exact test : *p* > 0.999, effect size odds ratio = 0.879). There was not significant different on prevalence of postoperative pain after 1 week and after at least 18 months between single-visit and multiple-visit treatment.

The chairside time (mean ± SD) for single-visit and multiple-visit endodontic treatment were 62.0 ± 23.5 min and 92.9 ± 37.8 min, respectively (mean difference = −30.9, 95 % CI: −39.4 to −22.4, effect size d = −0.996, *p* < 0.001). Results in the final model of multi-way ANCOVA after backward elimination showed the chairside time was reduced by single-visit treatment, use of magnifying loupe, treatment on a single-canal tooth and/or treatment on a non-vital tooth (Table 3).

**Table 3:** The effects of treatment visit and other variables on chairside time

Variable	Estimate	95 % CI	*p*-value
Single-visit treatment	−27.83	−33.80 to −21.87	<0.001
Use of magnifying loupe	−24.48	−30.47 to −18.50	<0.001
Single-canal tooth	−33.51	−39.57 to −27.45	<0.001
Vital tooth	9.40	1.44 to 17.36	0.021

(Final multi-way ANOVA model) (*n* = 220)

R^2^ = 0.599, Adjusted R^2^ = 0.592

Among the 117 teeth received single-visit endodontic treatment, 104 did not have signs and symptoms suggesting failure at the final evaluation. The success rate was thus 88.9 % (Table 4). There were 13 teeth (11.1 %) classified as failures, one tooth (0.9 %) was classified based on clinical criteria alone, 12 (10.3 %) were due to the presence of periapical radiolucency in the evaluation radiographs alone and none of the case was classified so by both the clinical and radiograph criteria. Among the 103 teeth received multiple-visit endodontic treatment, 90 teeth (87.4 %) were classified as success. Two teeth (1.9 %) were classified as failure based on both clinical and radiographic criteria, on tooth (1.0 %) on clinical criteria only, and 10 teeth (9.7 %) on radiographic criteria.

**Table 4:** Clinical and radiographic status of the teeth at the final evaluation

Treatment group	Single-visit	Multiple-visit	All
	*n* = 117 (%)	*n* = 103 (%)	*n* = 220 (%)
Successful			
No clinical or radiographic failure	*n* = 104 (88.9 %)	*n* = 90 (87 %)	*n* = 194 (88 %)
Failure			
(a) Both clinical and radiograph failure	*n* = 0 (0 %)	*n* = 2 (1.9 %)	*n* = 2 (0.9 %)
(b) Clinical failure (radiograph not classified)	*n* = 1 (0.9 %)	*n* = 1 (1.0 %)	*n* = 2 (0.9 %)
(c) Radiolucent area present, no clinical sign	*n* = 12 (10.3 %)	*n* = 10 (9.7 %)	*n* = 22 (10.0 %)
Total (a) + (b) + (c)	*n* = 13 (11.1 %)	*n* = 13 (12.6 %)	*n* = 26 (11.8 %)

## Discussion

A literature review found the dropout rate in longitudinal clinical studies could be 50 % and was a major source of error [17]. In this study, telephone reminders for follow-up examinations and individual, detailed preoperative explanations were provided to minimise the dropout rate. From the telephone communications, we found some patients considered their teeth had no problems and were not willing to come back for review. There was difference in gender distribution between groups. This could be a source of unknown bias in this study. The dropout rate was 14.1 %, which was considered acceptable when compared with other studies [18].

This study achieved the minimal required number of participants, but the sample was not very large. Therefore, the power of the statistical test used in this study was not high. A larger sample size would have given a higher power in the statistical analysis, but this is often difficult to achieve in clinical practice, where the number of eligible patents are limited [12]. A multi-centre clinical trial could be an option, but it requires more resources. Some researches might suggest a meta-analysis, but such results should be interpreted with caution since the protocol for endodontic treatment and the method for evaluation of treatment outcomes often vary between studies.

The treatment outcome was categorised as a success in this study when the treated tooth had no signs or symptoms in both clinical and radiographic assessments [12, 19]. A review period of at least 18 months was adopted. Pirani and colleagues suggested radiographic evaluation 6–9 months after treatment was an early prognostic tool to determine success [20]. An incomplete healing with a reduced size of apical radiolucency was regarded as a ‘questionable success’ in some studies [10, 11] but was considered as a failure in this study. Studies reported profound radiographic healing often be found after 3 months and mostly within 2 years [21, 22]. In this study, the average review period of the treated teeth was about 30 months, which was adequate for assessment of radiographic healing after endodontic treatment.

According to the results, the first null hypothesis of no differences in the success rate between single-visit and multiple-visit primary non-surgical endodontic treatment was accepted in this study. Moreover, the success rates of both treatment groups were high (88.9 and 87.4 %, respectively). The difference was small and had limited clinical implications. Some dentists believe performing multiple-visit treatment has a higher success rate than single visit [5]. This belief, however, was not substantiated according to the results of this study. The success rate of single-visit endodontic treatment in this study was very similar to another study reported by Field and his co-workers [23], though there was a slight different in the endodontic treatment protocol. In this study, the reasons for the tooth lost (extraction) were tooth fracture and secondary caries. There were only three teeth lost over the study period. The percentage is low and was not considered a significant outcome to be discussed in this study.

The obturation method used in this study was the core-carrier technique, which is different from previous studies that used conventional cold lateral condensation. However, a literature review found no difference in success rate between the cold lateral condensation and core-carrier obturation technique [1]. The core-carrier technique is simple and quick and has become a common obturation technique for general dentists [12]. In this study, the success rate of endodontic treatment was similar to other studies using a core-carrier obturation system [12, 24].

Researchers reported teeth with preoperative radiolucency had a lower chance of success [22, 25, 26]. Nair suggested apical periodontitis was a local tissue destruction resulting from the loss of balance between host responses and the presence of a microbial infection originating from the root canal system [27]. At present, a non-surgical endodontic technique that can ensure complete resolution of apical periodontitis is lacking. Failures still occur even though contemporary endodontic techniques with the highest standards and the most careful procedures are used. There might be unknown factors that influence disinfection of inflamed periapical tissue and, hence, the post-treatment healing of a lesion [27]. This provided explanation to a lower success rate for teeth with apical periodontitis than teeth without apical periodontitis in this study.

This study found no significant difference in postoperative pain between the single-visit and multiple-visit treatment groups. Therefore, the second null hypothesis was accepted. The common perception of dentists that multiple-visit endodontic treatment can reduce postoperative pain was therefore not validated [5]. Practically, quite a number of dentists who subjectively preferred multiple-visit treatment on account of the better success rate and reduced postoperative pain were not justified by this study [6].

The third null hypothesis—that there would be no difference in the chairside time used for single-visit and multiple-visit endodontic treatment—was rejected. Better recall of root morphology, established coronal access and reduction of repeated procedures such as application of rubber dam isolation and local anaesthetic allow dentists to shorten the total chairside time. The chairside time was significantly shorter with the use of a magnifying loupe and treatment on single-canal teeth; these findings concurred with another recent study [28]. From the time management point of view, patients could benefit from single-visit treatment. This may be desirable for anxious patients in need of sedation, those who are medically compromised or those who have special needs, hoping for reduced stresses built up prior to a dental visit and reduced treatment-associated risks [29]. Moreover, a single treatment shortens the time for oral rehabilitation because the tooth can be restored to function sooner.

This study found endodontic treatment success was not related to patients’ age or gender. This finding was in agreement with previous studies [23, 30]. However, this study found maxillary teeth showed a better chance of healing than mandibular teeth. This is either not supported by some previous studies [23, 30] or not investigated in other studies [1]. The distribution of the location of the treated teeth depended on the teeth that required endodontic treatment and, therefore, could not be randomised in this study. There were more maxillary teeth (63 %) and less mandibular teeth, especially mandibular anterior teeth, which required endodontic treatment. The tooth status, extent of caries destruction and prognosis were not recorded in this study. Therefore, whether these factors could affect the treatment outcome remains unknown. Contrary to the results of this study, Huumonen and Orstavik reported maxillary lateral incisors showed the poorest healing rate [21]. The great majority of the teeth with failure were posterior teeth. This finding was in agreement with several studies [22–24]. The difference might be explained by the root morphology, multiple canals and greater complexity [1].

Using periapical radiographs for assessment of success in endodontic treatment is a common practice. One of the problems with this assessment method is the reproducibility of the assessment results [31]. The inter- and intra-observer variations were not high in this study. The reviewers were experienced clinicians, and they received training on radiographic assessment on 50 radiographs. These radiographs were chosen for training purposes. They reflected different treatment outcomes and were not selected from the patients in this study.

Complete re-establishment of normal structure might not occur for all cases under non-surgical endodontic treatment [32, 33]. Therefore, there is no consensus whether complete periapical healing is a must in the success of endodontic treatment. Halse and Molven questioned the persistent apical radiolucency over 20 years as absolute failure [34]. They suggested there could be incomplete reformation of apical morphology or progress in healing due to over-extrusion of endodontic obturation materials. In this study, three-quarter of the failure cases were incomplete healing. Some clinicians have suggested that cone-beam computed tomography (CBCT) is preferred over periapical radiograph to evaluate treatment outcomes [17]. Cheung and co-workers concluded that there was a significant difference between CBCT and intra-oral radiography on periapical health status, especially on maxillary teeth [35]. CBCT should be justified individually based upon an inadequate amount of information gained by appropriate normal radiographs to reduce radiation doses [36]. There is no doubt that CBCT produces better imaging to improve the validity of the assessment of periapical bone healing after endodontic treatment [37]. However, CBCT requires larger doses of irradiation and, therefore, should not be the standard assessment method for research purposes.

This study reflected that single-visit non-surgical primary endodontic treatment could be performed by general dentists with a reasonable success rate. The paramount consideration for general practitioners is case selection. Those complicated and challenging cases including calcified canal, bifurcated canal and additional canal that could not be located without high-power magnification should be referred to an endodontic specialist for a better success rate. A preoperative discussion between dentist and patient should be done first and foremost, for the patient’s benefit, on the outcome of non-surgical endodontic treatment [29].

## Conclusions

In this study, the success rate of single-visit and multiple-visit endodontic treatments had no significant difference. There was also no statistical difference in the prevalence of postoperative pain between two treatment groups. The success rate was lower for mandibular teeth and in the presence of apical periodontitis. The chairside time for single-visit treatment was shorter than multiple-visit treatment.
